# Contrast-induced encephalopathy after an embolization procedure for a cerebral aneurysm in a female with subarachnoid hemorrhage: a case report

**DOI:** 10.1186/s12883-024-03530-5

**Published:** 2024-01-23

**Authors:** Jing Ren, Yinhua Ge, Ruyi Wen, Yong Zhang, Jun Shen, Wenjun Chen

**Affiliations:** 1 Department of Neurology, The Second Peoples Hospital of HuiShan, Wuxi, 214000 Jiangsu China; 2 Department of Radiology, The Second Peoples Hospital of HuiShan, Wuxi, 214000 Jiangsu China

**Keywords:** Contrast-induced encephalopathy, Aneurysm embolization, Computer tomography, Digital subtraction angiography, Case report

## Abstract

**Background:**

Contrast-induced encephalopathy (CIE) is a rare complication during or after angiography, usually transient and reversible. CIE diagnosis is challenging due to the absence of no formal diagnostic criteria. CIE can mimic stroke symptoms, including visual disturbances, seizures, confusion, coma, and focal neurological deficits. This case reports neurological deficit reversal in a CIE patient due to the embolization of an intracranial aneurysm, the second angiographic procedure in six days.

**Case presentation:**

A 77-year-old woman was admitted to the hospital for headaches. The cerebral computed tomography (CT) scan indicated a subarachnoid hemorrhage. The first digital subtraction angiography (DSA) identified an aneurysm of 4 mm ∗ 3 mm in size in the M1 segment of the right middle cerebral artery (MCA). Then, embolization surgery was performed for the cerebral aneurysm, which was successful. However, the patient had post-operative headaches, slurred speech, epilepsy, limb weakness, and delirium post-procedure. The non-contrast cerebral CT indicated widespread edema in the right cerebral hemisphere. The patient was diagnosed with CIE and treated with symptomatic supportive therapy. Eventually, the patient’s neurological deficits and cerebral edema improved significantly.

**Conclusions:**

The current case emphasized the importance of early diagnosis and symptomatic treatment of CIE. Thus, CIE should be the first consideration during the differential diagnosis of a patient having acute neurological impairment after repeated DSA.

**Supplementary Information:**

The online version contains supplementary material available at 10.1186/s12883-024-03530-5.

## Background

Contrast-induced encephalopathy (CIE) is a rare complication of angiography. It manifests different neurological symptoms, such as visual disturbances, seizures, confusion, coma, and focal neurological deficits [1]. Previous studies have indicated that most patients recover completely within 48–72 h without neurological deficits. However, there are rare cases of irreversible or even fatal CIE [1–3] without formal diagnostic criteria. At present, due to the differences in clinical presentations, neuroimaging and reversible processes are critical in differential diagnosis. The current report describes the reversal of severe neurological impairment in a patient suffering from subarachnoid hemorrhage who was diagnosed with CIE after an intracranial aneurysm embolization, her second DSA procedure.

## Case presentation

**Fig. 1:**
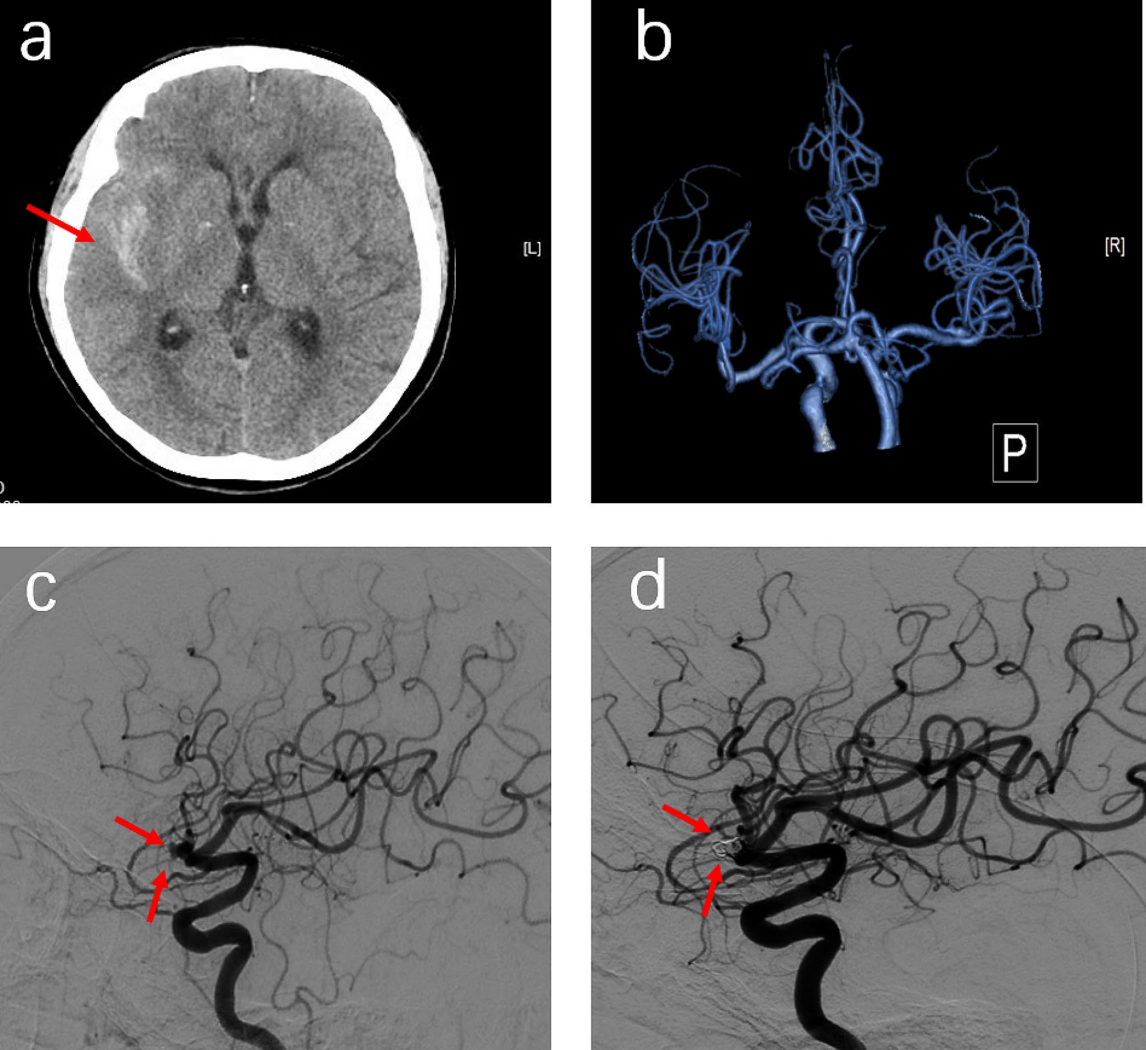
The imaging examinations before CIE. (**a**) Brain CT on the first day depicted a subarachnoid hemorrhage. (**b**) CTA detected no evident aneurysm or vascular malformation. (**c**) DSA revealed an aneurysm of 4 mm ∗ 3 mm in size in the M1 segment of the right MCA. (**d**) DSA demonstrated a coiling aneurysm embolization

**Fig. 2:**
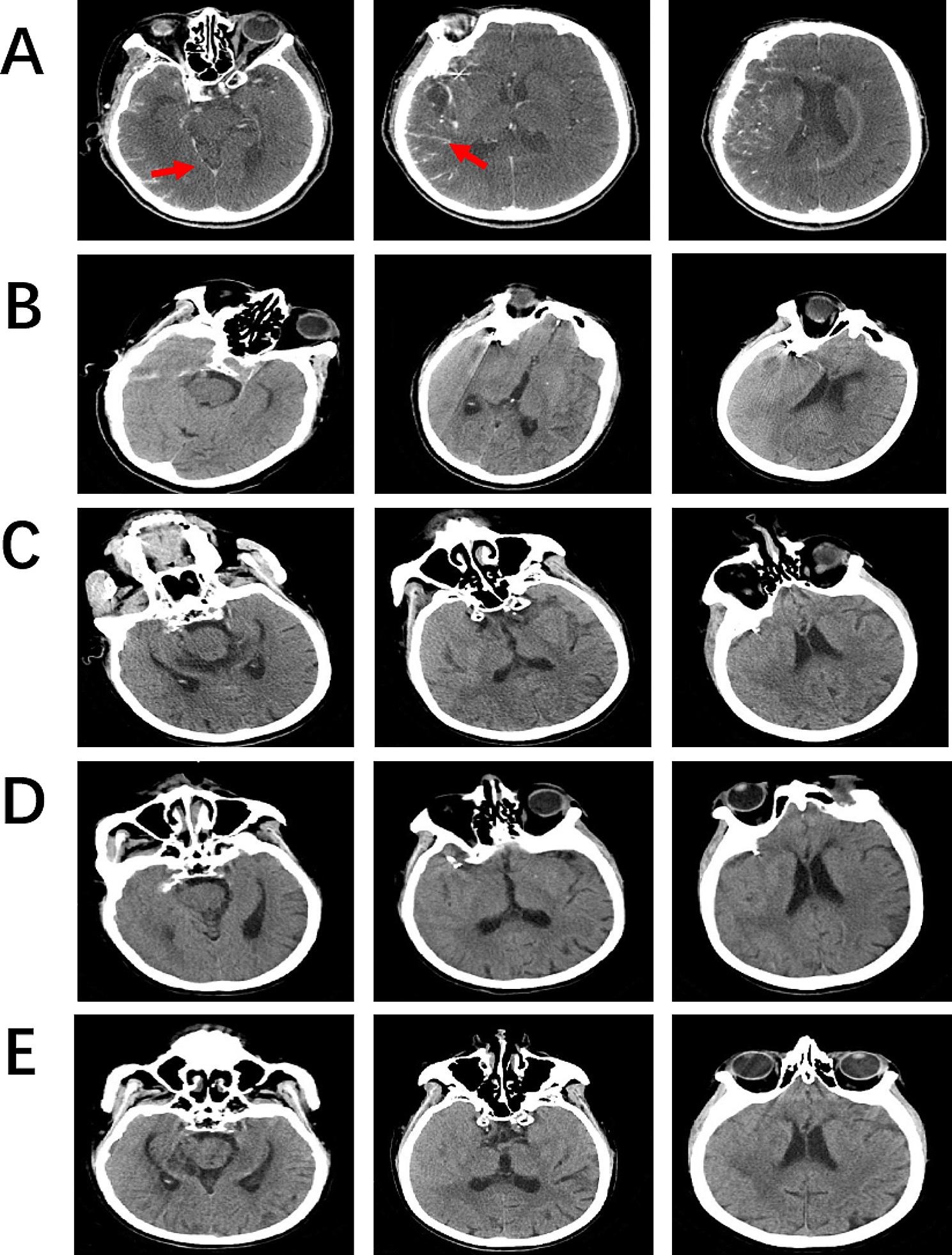
Brain CT scans of the CIE patient. (**A**) The Xper-CT scan was performed immediately post-operation. (**B**) The brain CT scan was performed after 7 h of the procedure. (**C**) The brain CT scan was performed the following day post-procedure. (**D**) The brain CT scan was performed on the 4th day post-procedure. (**E**) The brain CT scan was performed on the 6th day post-procedure

A 74-year-old woman was admitted to our hospital due to headaches, nausea, and vomiting for three days. The patient possessed no history of hypertension, coronary artery disease, or diabetes. Her laboratory profile, including renal and hepatic function, was normal. The physical examination indicated a positive meningeal irritation sign. A cranial computed tomography (CT) scan of the brain depicted a subarachnoid hemorrhage in the right cerebral hemisphere (Fig. 1a). The patient’s symptoms had entirely resolved post-treatment with nimodipine and mannitol. The patient received a computed tomography angiography (CTA) examination for further assessment. The results showed no evident aneurysm or vascular malformation (Fig. 1b). The patient underwent digital subtraction angiography (DSA) under local anesthesia with a total of 120 ml of ioversol to identify the cause. DSA revealed a 4 mm ∗ 3 mm aneurysm inside the M1 segment of the right middle cerebral artery (MCA) (Fig. 1c). No complications were observed during or after the procedure. After six days, the patient received a therapeutic embolization procedure for a cerebral aneurysm with 120 ml of ioversol under general anesthesia (Fig. 1d). The Xper-CT was performed immediately after the process, showing cortical and subarachnoid enhancement (Fig. 2A). Later, the patient returned safely to the wards post-surgery. Approximately seven hours post-procedure, the patient manifested mild bearable headaches. This is followed by slurred speech, left-hand muscle strength loss (level 0/5), dysphoria, tongue deviation, and a positive left Babinski sign within four hours. An immediate brain CT scan detected edema within the right cerebral hemisphere involving the parietal and temporal lobes without hemorrhage (Fig. 2B). Since DSA did not reveal cerebrovascular stenosis and Xper-CT revealed enhanced cortical and subarachnoid, the patient was suspected of CIE. We used 5 mg of dexamethasone and 250 ml of glycerol fructose to decrease brain edema. Meanwhile, glucose sodium chloride helped eliminate the contrast agent. On the following day post-procedure, there was no improvement in neurologic impairment symptoms, although the CT revealed an improvement in right brain edema (Fig. 2C). On the third day, the patient’s symptoms further deteriorated, with several generalized tonic-clonic seizures. Despite being promptly treated with 10 mg diazepam through intravenous injection and 0.5 g sodium valproate extended-release oral tablets, the symptoms were poorly controlled. Then, the patient was transferred to the intensive care unit for further treatment, where she received a head CT scan the next day (Fig. 2D). Midazolam was continuously infusioned to treat epilepsy. Simultaneously, we initiated intensive dehydration therapy. Human serum albumin, methylprednisolone, and glycerol fructose helped reduce intracranial pressure. Thus, all neurologic deficits and brain edema (Fig. 2E) improved significantly on postoperative Day 6 while she was transferred back into the general ward. The patient was discharged on the 8th day after the second DSA with only a tongue deviation.

## Discussion and conclusions

CIE is a rare and generally reversible complication during or after angiography. Based on the previous study, most patients reported a good prognosis, while a few cases had a permanent neurological deficit, even death [1, 3]. With the large-scale angiography application, more CIE cases have been demonstrated recently.

The early diagnosis of CIE is challenging since it mimics cerebrovascular accidents. CIE lacks unified diagnostic criteria and is an exclusion diagnosis, depending on clinical course and neuroimaging findings. The typical CT findings for CIE depict anomalous cerebral edema and contrast enhancement inside the subarachnoid space and striatum [4]. Abnormal MRI has indicated the hyperintensity and gyrus swelling on the T2 fluid-attenuated inversion recovery (FLAIR) image and diffusion-weighted imaging (DWI) in previous studies [5]. In this case, our report showed that the patient exhibited distal limb weakness, slurred speech, tongue deviation, and consciousness disorders post-neuro-interventional procedures. The multiple CT scans indicate no infarction or hemorrhage. The CIE could have been the leading cause of these clinical manifestations. In six days, the patient recovered with reduced intracranial pressure, corticosteroids, and anti-seizure therapy.

However, the pathophysiological mechanism behind CIE remains unclear. The widely accepted theory is the blood-brain barrier (BBB) disruption. According to previous reports, the contrast agent breaks the BBB while entering the cortex and subarachnoid [6]. Contrast agents can generally be classified as either ionic or nonionic based on their ability to ionize in solution. Additionally, they could be categorized as hypertonic, hypo-hyperosmolar, or isotonic, depending on their osmolality relative to human plasma. The nonionic contrast agent is widely used due to its significant safety than the ionic type. The blood-brain barrier is impermeable to contrast material under normal conditions. The high-osmolality contrast medium could open the normal endothelial cell-tight BBB junctions. Previous articles depicted that various contrast mediums can induce CIE despite their permeability or ionic states [5, 7]. In this case, the patient used the second-generation iso-osmolar nonionic contrast agent ioversol. Animal studies indicated the chemical and/or physical effects of contrast media directly damage the BBB [8]. Therefore, the CIE mechanism is controversial and requires further investigation.

CIE risk factors were explored in previous studies, with stroke history and renal failure recognized as the main factors [9]. Moreover, age, gender, contrast medium type, contrast medium dose, and hypertension were also attributed to CIE occurrence [10]. Dysfunctional kidneys decrease the clearance rate of contrast medium, accumulating the osmolality and neurotoxicity of contrast. Stroke may disrupt the BBB within the same or adjacent vascular territory, promoting contrast leakage into broader brain regions and causing tissue reactions and brain edema. A recently published article indicated that general anesthesia could be a potential CIE risk factor [11]. The patient had no renal failure or chronic disease history in the present case. However, she suffered a subarachnoid hemorrhage ten days before the procedure, facilitating the contrast agent outflow within the brain parenchyma. Moreover, the CIE did not happen in the first DSA but in the second DSA under general anesthesia. Some of the anesthetic drugs could be associated with the elevated occurrence of CIE. Furthermore, the second-generation iso-osmolar nonionic contrast agent was used twice in the patient in six days. A safer third-generation iso-osmolar nonionic contrast agent should be chosen during later procedures.

There are no guidelines to treat CIE. A multidisciplinary approach and close monitoring of the patient’s neurological function post-surgery are required. Treatment plans include promoting contrast clearance, decreasing cerebral edema, anti-inflammatory therapy, and symptomatic supportive therapy. Steroids and mannitol can relieve cerebral edema [12]. Besides, continuous renal replacement therapy and blood purification could potentially treat severe CIE patients [13]. Early detection and timely CIE treatment can prevent permanent neurologic damage, leading to a good prognosis. In this article, the patient was treated with hydration, intravenous dexamethasone, mannitol, and antiepileptic drugs, progressively improving her neurological deficits within five days.

Long-term MCA aneurysm recurrences occurred in nearly one-quarter of cases, necessitating retreatment in one out of 10 patients [14]. It is necessary to countercheck cerebrovascular angiography and reevaluate cerebral vascular. There is no formal expert consensus for CIE patients to recommend if they can undergo an angiography procedure again, making it challenging for experienced doctors.

Thus, CIE may not happen in the first DSA procedure, but recurrent DSA procedures elevate the CIE risk. Therefore, CIE should be taken into consideration while differentially diagnosing a patient with acute neurological impairments after repeated DSA. This case emphasizes the importance of early diagnosis and symptomatic treatment to decrease CIE incidence and mitigate its disability. Doctors performing cerebral vascular angiography and interventions should consider the potential harmful effects. In short, further investigations are required to ascertain whether the number of cerebral angiographies and anesthesia methods could be a CIE risk factor.

### Electronic supplementary material

Below is the link to the electronic supplementary material.

Supplementary Material 1

## Data Availability

Not applicable.

## Abbreviations

BBB: Blood-brain barrier
CIE: Contrast-induced encephalopathy
CT: Computed tomography
DWI: Diffusion-weighted imaging
DSA: Digital subtraction angiography
FLAIR: Fluid-attenuated inversion recovery
MCA: Middle cerebral artery
MRA: Magnetic resonance angiography
MRI: Magnetic resonance imaging
