# Supplementary material for: Contrast-induced encephalopathy after an embolization procedure for a cerebral aneurysm in a female with subarachnoid hemorrhage: a case report
Source: BMC Neurol. 2024 Jan 23;24:38. doi: 10.1186/s12883-024-03530-5 (PMC10804565; doi:10.1186/s12883-024-03530-5)
Supplement: Supplementary file 1 — Supplementary Material 1 [file 12883_2024_3530_MOESM1_ESM.pdf]

CARE case report guidelines

CARE Checklist of information to include when writing a case report

(cc) BY-NC-ND

Topic	Item	Checklist item description	Reported on Line
Title	1	The diagnosis or intervention of primary focus followed by the words "case report"	Line 2
Key Words	2	2 to 5 key words that identify diagnoses or interventions in this case report, including "case report"	Line 52-53
Abstract (no references)	3a	Introduction: What is unique about this case and what does it add to the scientific literature?	Line 35-37
	3b	Main symptoms and/or important clinical findings	Line 43-45
	3c	The main diagnoses, therapeutic interventions, and outcomes	Line 45-47
	3d	Conclusion—What is the main "take-away" lesson(s) from this case?	Line 48-50
Introduction	4	One or two paragraphs summarizing why this case is unique (**may include references**)	Line 61-70
Patient Information	5a	De-identified patient specific information.	Line 73
	5b	Primary concerns and symptoms of the patient.	Line 73-74; Line 87-90
	5c	Medical, family, and psycho-social history including relevant genetic information	Line 74-75
	5d	Relevant past interventions withoutcomes	Line 77-78
Clinical Findings	6	Describe significant physical examination (PE) and important clinical findings.	Line 75-83; Line 88-92
Timeline	7	Historical and current information from this episode of care organized as a timeline	Line 85-108
Diagnostic Assessment	8a	Diagnostic testing (such as PE, laboratory testing, imaging, surveys).	Line 89-92
	8b	Diagnostic challenges (such as access to testing, financial, or cultural)	Line 92-93
	8c	Diagnosis (including other diagnoses considered)	Line 93
	8d	Prognosis (such as staging in oncology) where applicable	N/A
Therapeutic Intervention	9a	Types of therapeutic intervention (such as pharmacologic, surgical, preventive, self-care)	Line 93-95;Line 99-104
	9b	Administration of therapeutic intervention (such as dosage, strength, duration)	Line 93-94;Line 99-100
	9c	Changes in therapeutic intervention (with rationale)	Line 99-105
Follow-up and Outcomes	10a	Clinician and patient-assessed outcomes (if available)	Line 105-106
	10b	Important follow-up diagnostic and other test results	Line 107-108
	10c	Intervention adherence and tolerability (How was this assessed?)	N/A
	10d	Adverse and unanticipated events	N/A
Discussion	11a	A scientific discussion of the strengths AND limitations associated with this case report	Line 155-157; Line 165-169
	11b	Discussion of the relevant medical literature **with references**.	Line 111-175
	11c	The scientific rationale for any conclusions (including assessment of possible causes)	Line 136-141; Line 155-158
	11d	The primary "take-away" lessons of this case report (without references) in a one paragraph conclusion	Line 176-183
Patient Perspective	12	The patient should share their perspective in one to two paragraphs on the treatment(s) they received	N/A
Informed Consent	13	Did the patient give informed consent? Please provide if requested	Yes √ No ☐
